# A 3PN zygote can be transferred after PGT-A and embryo ploidy analysis: A case report of a healthy neonate

**DOI:** 10.5935/1518-0557.20250182

**Published:** 2026

**Authors:** Eliana Margarita Retamoso-Paz, Marisol Martínez-Martínez, Luz Sthefany Chavarro-Tello, Clara Inés Esteban-Pérez

**Affiliations:** 1 Department of Gynecology and Obstetrics. Universidad del Sinú EBZ. Cartagena, Colombia; 2 FIV Laboratory, Cecolfes. Cartagena, Colombia; 3 Reproductive Genomic. Invitrocell S.A.S. Bogotá, Colombia

**Keywords:** case report, 3PN embryo, ICSI, PGT-A, SNP, NGS

## Abstract

This case report describes a healthy live birth following in vitro fertilization (IVF) resulting from the transfer of a euploid embryo derived from a tri-pronuclear zygote (3PN), along with a review of related literature. The case occurred at a private IVF center. It involves a nulliparous 29-year-old woman with abnormal ovarian reserve and male factor infertility who underwent IVF. Preimplantation genetic testing for aneuploidy and noninvasive prenatal testing with cell-free fetal DNA indicated normal ploidy. This report provides evidence that transferring a euploid embryo from an abnormally fertilized oocyte (tri-pronuclear zygote) can lead to a clinical pregnancy and a healthy live birth.

## INTRODUCTION

Normal fertilization is marked by the presence of two pronuclei (PN) and two polar bodies (PB) in zygotes 16-20 hours after fertilization. Conditions that differ from these are considered abnormal (Guo *et al.*, 2024). The occurrence of 3PN zygotes in all pregnancies is estimated to be about 1-3%. The incidence ranges from 5.0% to 8.1% during in vitro fertilization (IVF) and from 2.5% to 6.2% in intracytoplasmic sperm injection (ICSI) (Mutia *et al.*, 2019).

The formation of multiple pronuclei in IVF mainly results from more than one sperm fertilizing the oocyte. However, the primary cause of polypronuclear formation after ICSI appears to be the oocyte’s inability to extrude the second polar body due to its failure to complete the second meiotic division after sperm injection. This then converts into a pronucleus (Matt *et al.*, 2004; Haddad *et al.*, 2021). Nonetheless, other mechanisms have been suggested, such as fertilization of a diploid oocyte by a haploid spermatozoon or fertilization of an oocyte by a diploid spermatozoon (Grigoryan *et al.*, 2019; Cermisoni *et al.*, 2024).

Generally, 3PN zygotes are discarded in clinical practice because they are believed to have a polyploid chromosomal makeup, and transferring these embryos carries a higher risk of miscarriage and/or molar pregnancy (Takahashi *et al.*, 2022). The very few fetuses that survive and are delivered often have severe congenital malformations (Chen *et al.*, 2022).

In this case report, we describe a successful birth of a healthy neonate from a woman whose transferred embryo originated from a 3PN zygote formed after ICSI, with normal chromosomal status confirmed by preimplantation genetic testing for aneuploidy (PGT-A).

## CASE REPORT

### Patient information

We present a case of a 29-year-old woman who has never given birth, seen by a gynecology specialist in March 2022, with dysuria as her only symptom; she planned to become pregnant within the following year.

### Medical History

Aside from recurrent cystitis treated by a urologist and a history of umbilical herniorrhaphy, the patient denies any other significant medical, allergic, traumatic, or toxicological issues. She is the youngest full-term daughter of a 40-year-old woman. Gynecological history includes menarche at age 13, with 28-day cycles; she has been living with her partner for over a year without using any contraception.

During the physical examination, she showed no specific signs; her body mass index was 22. An ultrasound revealed the right ovary had a volume of 10.5 cm^3^, and the left ovary had a volume of 2.3 cm^3^, each with an antral follicle count (AFC) of 3. The initial diagnoses were primary infertility and an abnormal ovarian reserve (Ferraretti *et al.*, 2011).

### Complementary tests

Anti-Mullerian hormone (AMH) with a low level concentration: 0.04 ng/mL, and a sperm analysis showing oligo-asthenic-teratozoospermia with necrozoospermia.

### Intervention

Management with IVF/ICSI was recommended due to male factor with poor prognosis for ART (Esteves *et al.*, 2019). Her first cycle used a mild stimulation protocol with FSHr (Bemfola^®^ 225U) for 6 days plus Clomiphene (Zimaquin^®^ 100mg) for 5 days. The final oocyte maturation was triggered with 5000 IU of hCG. Oocyte retrieval was performed via transvaginal follicular aspiration under ultrasonographic guidance, resulting in the retrieval and cryopreservation of two mature oocytes.

A second cycle was performed using the same doses of FSHr for 8 days. Three mature oocytes were retrieved and cryopreserved. The five oocytes collected from both cycles were inseminated via IVF/ICSI, but no embryos resulted for transfer.

The third cycle was conducted according to the specifications in Table 1. First, a fresh embryo transfer was performed using embryo #5, which was unsuccessful. Next, a vitrified-warmed embryo transfer was carried out using embryo #3, following an artificial endometrial preparation cycle with transdermal estradiol 2 mg/day (0.06% estradiol gel, Ginoderm^®^) and oral equine conjugated estrogens 0.0625 mg/day (Estermax^®^ tablets). The endometrial thickness reached 12 mm; as a result, luteal phase support was initiated with vaginal progesterone 600 mg/day (Jarit^®^). Twelve days after embryo transfer, the quantitative β-hCG test was negative.

**Table 1:** Third Cycle stimulation.

Stimulation	Trigger	Oocyte	Mature	Zygote	Day 3	Day 5	Technique	Result
6 Days: FSHr (Bemfola^®^225U) 5 Days: Clomiphene (Zimaquin^®^ 100mg)	HCG 5000UI	**1**	**MII**	3PN2PB	8BL	4AA	Cryopreserved/warmed ET	Pregnancy
		**2**	**MII**	0PN2PB	6BL	Arrested	N/A	N/A
		**3**	**MII**	2PN2PB	8BL	4BB	Cryopreserved/warmed ET	Failure
		**4**	**MII**	2PN2PB	8BL	Arrested	N/A	N/A
		**5**	**MII**	2PN2PB	8BL	N/A	Fresh ET	Failure
		**6**	**GV**	N/A	N/A	N/A	N/A	N/A

**Figure 1:**
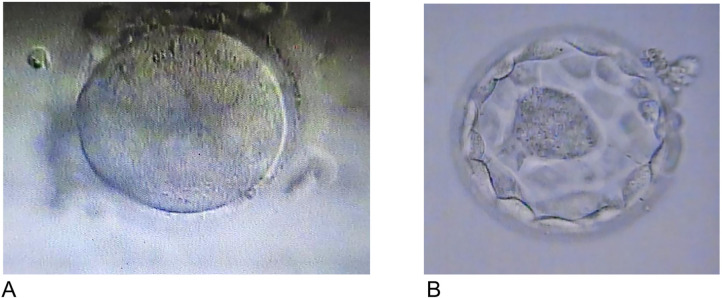
A. Zygote No. 1: 3 PN. B Embryo No. 1: Blastocyst 4AA.

With no other option, we decided to warm the last vitrified embryo (3PN, see Fig. 1) and perform preimplantation genetic testing for aneuploidy (PGT-A) from a trophectoderm biopsy and ploidy analysis. The embryo was vitrified again, and biopsy samples were analyzed at Cooper Genomics labs using next-generation sequencing and heterozygous SNPs allele ratio determination, resulting in a Euploid and 1:1 allele-diploid outcome.

Once we had the result of the euploid embryo, it was warmed and transferred during a natural cycle endometrial preparation on LH +6 day. The luteal phase progesterone with micronized vaginal progesterone (600 mg/day) was started in the evening after frozen embryo transfer (progesterone administration was continued until 12 weeks of pregnancy). Twelve days after embryo transfer, a quantitative β-hCG test showed 1090 mIU/mL.

At 6 weeks and 6 days of pregnancy, transvaginal ultrasonography revealed one gestational sac in the uterus with a positive fetal heartbeat. At 13 weeks of pregnancy, maternal blood was taken for a genetic test analyzing circulating cell-free fetal DNA in plasma, confirming the absence of aneuploidies. Thereafter, her pregnancy proceeded without any adverse or unexpected events.

At 39 weeks of pregnancy, an elective cesarean section was performed due to external hemorrhoids with a risk of thrombosis, and a girl was born in good overall condition weighing 3440 grams. Postpartum, the mother and the newborn recovered without any complications.

## DISCUSSION

In IVF procedures, many parameters are used to select embryo quality for transfer, such as embryo morphology, cell division rate, pronuclear morphology, and progression to the blastocyst stage (Balaban *et al.*, 2001). In our case, the patient had a 3PN zygote; it is well known that when a zygote has one, three, or more PNs, it is considered abnormally fertilized, and therefore they are usually discarded due to the increased risk of abnormal ploidy (Takahashi *et al.*, 2022; Canon *et al.*, 2023).

It is important to note that, although many studies have shown that approximately 10%-31% of 3PN zygotes reach the blastocyst stage (Sathananthan *et al.*, 1999; Fan *et al.*, 2013; van de Werken *et al.*, 2015; Yao *et al.*, 2016; Yoder *et al.*, 2021), this does not mean they are genetically normal; rather, it likely indicates that the first cleavage is the critical stage in these zygotes (Yalçınkaya *et al.*, 2016), which is why performing PGT-A on days 5 or 6 is often recommended, as we did with our patient.

The study by Mutia *et al*., showed that a significant portion of 3PN zygotes from ICSI-IVF had normal chromosomes (33.3%). The chromosomal abnormalities in the sample population were mainly due to triploidy, mosaicism, and aneuploidy, with respective frequencies of 43.3%, 13.4%, and 10% (Mutia *et al.*, 2019). According to the study by Canon *et al*., a third pronucleus can also develop as smaller than the typical 3PN, known as “micro 3PN.” When the authors performed PGT-A analyses on these micro 3PN-derived embryos, they found that 27.5% were euploid (Canon *et al.*, 2023).

An abnormal PN count is not, in fact, a direct predictor of the ploidy state of a zygote (Mutia *et al.*, 2019; Canon *et al.*, 2023). As shown in our case report, a euploid embryo reported as 46, XX after PGT-A testing and ploidy determination subsequently resulted in the birth of a healthy neonate.

This indicates that 3PN zygotes should not be automatically discarded during IVF procedures when no other embryos are available for transfer. Patients should be fully informed about PGT-A as an option to confirm chromosome normality and ploidy on days 5 or 6 after fertilization (blastocyst stage).

The limitation of our study was the patient’s refusal to perform a karyotype analysis on the newborn to confirm euploidy.

## CONCLUSION

In this case report, we present evidence that a healthy, euploid neonate can develop from a blastocyst derived from a 3PN zygote after PGT-A and ploidy analysis. Abnormally fertilized oocytes may be clinically usable when no other embryos are available for the patient; in such cases, embryo transfer can be performed, potentially resulting in a healthy newborn.

## PATIENT PERSPECTIVE

If I had to describe my experience during this entire infertility journey, I would call it a roller coaster of emotions. Knowing that my greatest dream of becoming a mother was in the hands of science motivated me to believe in every step throughout this treatment. During this process, three transfers were performed; two of them without success, and the final one with a blastocyst that resulted in pregnancy. Watching life develop from the smallest particles to seeing a positive pregnancy test was very exciting. Today, I can say that everything was successful: my body responded well to effective and painless stimuli, the transfer was flawless, and each step was done on time. Thank you, Cecolfes Cartagena, for this miracle.

## Abbreviations:

IVF: in vitro fertilization, 3PN: tri-pronuclear zygote, PN: pronuclear, PB: polar bodies, ICSI: intracytoplasmic sperm injection, PGT-A: preimplantation genetic testing for aneuploidy, AFC: antral follicle count, AMH: Anti-Müllerian hormone, MII: metaphase II, GV: germinal vesicle, BL: blastomeres, ET: embryo transfer, SNPs: single nucleotide polymorphisms, NGS: next-generation sequencing, ART: assisted reproduction techniques, FSHr: follicle-stimulating hormone recombinant, hCG: human chorionic gonadotropin, LH: luteinizing hormone, DNA: deoxyribonucleic acid.
